# Identification of *Enterococcus faecalis* enzymes with azoreductases and/or nitroreductase activity

**DOI:** 10.1186/s12866-017-1033-3

**Published:** 2017-05-25

**Authors:** Valérie Chalansonnet, Claire Mercier, Sylvain Orenga, Christophe Gilbert

**Affiliations:** 1 bioMérieux, 3 route de port Michaud, 38390 La Balme les Grottes, France; 2 CIRI, International Center for Infectiology Research, Legionella pathogenesis group, Université de Lyon, Lyon, France; 3 INSERM, U1111, Lyon, France; 4 Ecole Normale Supérieure de Lyon, F-69364 Lyon, France; 5 Université Lyon 1, F-69622 Lyon, France; 6 CNRS, UMR5308, Lyon, France

**Keywords:** Azoreductases, Nitroreductases, *Enterococcus faecalis*

## Abstract

**Background:**

Nitroreductases, NAD(P)H dependent flavoenzymes, are found in most of bacterial species. Even if *Enterococcus faecalis* strains seems to present such activity because of their sensitivity to nitrofurans, no enzyme has been described.

Nitroreductases were separated of others reductases due to their capacity to reduce nitro compounds. They are further classified based on their preference in cofactor: NADH and/or NADPH. However, recently, azoreductases have been studied for their strong activity on nitro compounds, especially nitro pro-drugs. This result suggests a crossing in azo and nitro reductase activities. For the moment, no nitroreductase was demonstrated to possess azoreductase activity. But due to sequence divergence and activity specificity linked to substrates, activity prediction is not evident and biochemical characterisation remains necessary.

Identifying enzymes active on these two classes of compounds: azo and nitro is of interest to consider a common physiological role.

**Results:**

Four putative nitroreductases, EF0404, EF0648, EF0655 and EF1181 from *Enterococcus faecalis* V583 were overexpressed as his-tagged recombinant proteins in *Escherichia coli* and purified following a native or a denaturing/renaturing protocol. EF0648, EF0655 and EF1181 showed nitroreductase activity and their cofactor preferences were in agreement with their protein sequence phylogeny. EF0404 showed both nitroreductase and azoreductase activity. Interestingly, the biochemical characteristics (substrate and cofactor specificity) of EF0404 resembled the properties of the known azoreductase AzoA. But its sequence matched within nitroreductase group, the same as EF0648.

**Conclusions:**

We here demonstrate nitroreductase activity of the putative reductases identified in the *Enterococcus faecalis* V583 genome. We identified the first nitroreductase able to reduce directly an azo compound, while its protein sequence is close to others nitroreductases. Consequently, it highlights the difficulty in classifying these enzymes solely on the basis of protein sequence alignment and hereby the necessity to experimentally demonstrate the activity. The results provide additional data to consider a broader functionality of these reductases.

## Background

Oxygen-insensitive nitroreductases are a group of flavoenzymes, belonging to oxido-reductases, that are able to reduce nitro compounds depending on nicotinamide adenine dinucleotide availability (NAD(P)H) [1]. They catalyze the sequential reduction of nitro groups through the addition of electron pairs from NAD(P)H to produce nitroso -, hydroxylamino- and eventually amino-compounds [2].

Nitroreductases have been isolated from a large number of bacterial species [3–7]. In fact, they are considered for biodegradation of nitroaromatic pollutants in particular explosives such as 2, 4, 6-trinitrotoluene (TNT) [8]. Moreover, in anti-cancer strategy, nitroreductases are one of the most studied candidates for gene-directed enzyme-prodrug therapy [9]. Due to these potential applications, nitroreductases have been well studied in enteric bacteria, except for *Enterococcus faecalis*, a Gram positive opportunistic pathogen present in the intestine of a variety of mammals. For this species, nitroreductase activity has never been proven and no nitroreductase enzyme has as yet been characterised. Nitroreductase activity in *E. faecalis* can be hypothesised from the observation that *E. faecalis* strains are usually sensitive to nitrofurans, antibiotics that are often used in case of urinary tract infections and which have retained value due to the expansion of resistance to *ß-*lactams [10]. Since the antimicrobial effect of this class of molecules is mostly mediated by reduced products generated through bacterial nitroreductase activity, the presence of nitroreductases in *E. faecalis* can be expected. While it appears useful to identify them for potential improvements of such applications.

A phylogenetic analysis allows classification of oxygen-insensitive nitroreductases into two groups. Group A nitroreductases are usually NADPH-dependent whereas group B nitroreductases can use both NADH and NADPH as electron donors [8]. Despite this classification, nitroreductases physiological substrates and roles remain unclear. In *E. coli*, *nfsA* expression is depending on oxidative stress response mediated by SoxRS [11]. This suggests an involvement in cell response to toxic compounds exposure. Furthermore, recent studies have demonstrated that azoreductases are able to reduce a larger set of compounds, including quinones and nitroaromatics, than their first known substrates: azo-compounds [6, 12]. This evidence suggests connections in between these reductases families.

In *E. faecalis*, only one azoreductase (AzoA) has been well characterised [13]. Azoreductases can also be classified on the basis of their cofactor use (NADH or NADPH) and prosthetic group dependence, covalent linkage of flavin mononucleotide (FMN) in particular [14, 15]. Type one and two are FMN-dependent azoreductases preferentially using NADH or NADPH, respectively. Type 3 enzymes are FMN independent azoreductases. The reduction of azo bonds occurs through a similar mechanism as the one for nitro reduction, a bi-bi ping pong mechanism enabling a two-electron transfer [16]. Therefore, there is an interest in similarities and differences between these enzymes, especially regarding their substrate specificities.

In this study, we aimed to confirm nitroreductase activity in *E. faecalis* strains and to identify the enzymes possibly involved. Based on genome annotations of *E. faecalis* V583 and protein sequence motif prediction, we selected four putative nitroreductases: EF0404, EF1181, EF0648 and EF0655. We cloned and purified these enzymes and tested their nitroreductase activity, FMN-dependence and cofactor preference. Taking into account that the reduction of nitro compounds by azoreductases has been previously demonstrated, we tested the nitroreductase activity of AzoA but also the azoreductase activity of the putative *E. faecalis* nitroreductases identified here*.*

## Methods

### Reagents

Oligonucleotides were synthesised by Life Technologies (Carlsbad, CA, US). Except otherwise mentioned, all other chemicals were supplied by Sigma-Aldrich (St. Louis, MO, US).

### Bacterial strains and plasmids

*E. faecalis* (EF) and *Escherichia coli* (EC) strains were selected from the bioMérieux strain collection. They were isolated from human, animal or food sources and originated from different geographic areas (Table 1). *E. faecalis* V583 was used as matrix for the amplification of putative reductases coding genes.

**Table 1:** Strains used in the study

Species	Collections	Code	Number
*Escherichia coli*	bioMérieux	EC	76.10.041
*Enterococcus faecalis*	bioMérieux	EF1	80.04.017
*Enterococcus faecalis*	bioMérieux – ATCC29212	EF2	83.11.066
*Enterococcus faecalis*	bioMérieux	EF3	95.01.009
*Enterococcus faecalis*	bioMérieux	EF4	98.06.158
*Enterococcus faecalis*	bioMérieux	EF5	00.08.222
*Enterococcus faecalis*	bioMérieux	EF6	04.05.001
*Enterococcus faecalis*	bioMérieux	EF7	07.06.031
*Enterococcus faecalis*	bioMérieux – ATCC700802	V583	95.07.074
*Escherichia coli*	bioMérieux – Stratagene	XL1Blue	13.02.209

*E. coli* XL1Blue (Stratagene, San Diego, US) was host for the modified pQE30 plasmids (Qiagen, Courtaboeuf, France) used for recombinant protein overexpression (Table 2).

**Table 2:** Plasmids constructed for the study

Name	Cloned gene	DNA extracted from
pQE30-azoA95	*azoA*	*Enterococcus faecalis* V583
pQE30-EF0404	*ef0404*	*Enterococcus faecalis* V583
pQE30-EF0648	*ef0648*	*Enterococcus faecalis* V583
pQE30-EF0655	*ef0655*	*Enterococcus faecalis* V583
pQE30-EF1181	*ef1181*	*Enterococcus faecalis* V583

### Bacterial nitroreductase activity testing

Eight *E. faecalis* strains and an *E. coli* strain as control, all part of bioMérieux strains collection were tested for their nitroreductase activity. For each strain, 100 μL of a 1 McFarland suspension was inoculated into 100 μL of Trypcase Soy broth (bioMérieux, France) containing 150 μM of 7-nitrocoumarin-3-carboxylic acid (7NCCA) [17] and incubated at 35 °C with shaking for 24 h. The bacterial reduction of this nitro substrate generates a fluorescent product (λ_ex_ = 365 nm, λ_em_ = 440 nm). Kinetic of nitroreduction was followed on an Infinite® M200 microplate reader (TECAN, Männedorf, Switzerland).

### In silico search of nitroreductases in the *E. faecalis* V583 genome sequence

The protein BLAST search was carried out on *E. faecalis* V583 published transcribed genome using two reference sequences: NfsA (NCBI reference sequence AAC73938.1.) and NfsB (AAC73679.1.), which are the two major nitroreductases in *E. coli* MG1655. As *E. coli* azoreductase AzoR displays nitroreductase activity [6, 18], a similar BLAST protein search was also performed using AzoR as the reference protein (AAC74494.1.).

### Phylogenetic data analyses

Sequence alignments and tree constructions were done using Geneious 7.1 (http://www.geneious.com, [19]). Protein sequences were compared using Muscle alignment. Trees were constructed using neighbour-joining method and out-grouped with the NQO1 sequence, a human quinone NADH dehydrogenase (AAB60701.1). The selected sequences all represented experimentally verified bacterial azoreductases and/or nitroreductases.

### Cloning of targeted genes

*E. faecalis* strain V583 DNA was used for amplification of putative nitroreductases coding genes. The plasmid pQE30 (Qiagen, Courtaboeuf, France) was used for cloning.

To obtain chromosomal DNA, *E. faecalis* cells were lysed in a solution containing Tris (0.1 M), EDTA (0.01 M) pH 8 and lysozyme (20 mg.ml^−1^) during 30 min at 37 °C followed by addition of proteinase K (1.4 mg.ml^−1^), RNase (1.4 mg.ml^−1^) and sarcosyl solution (2%). Incubation with slow shaking was continued for another hour at 37 °C. DNA was then extracted using a phenol/chloroform/isoamylalcohol mix (V/V/V; 25/24/1) (Roth, Karlsruhe, Germany) and chloroform/isoamylacohol (V/V; 24/1) before precipitation by cold ethanol (at 70% final concentration). The oligonucleotides used for gene amplification and cloning are listed in Table 3. PCR was carried out as described by Mercier et al. [18]. PCR products were analysed (5 μL aliquots) by agarose gel electrophoresis (1% agar in Tris-acetate-EDTA buffer) and further purified using the QIAquick purification kit (Qiagen, Courtaboeuf, France).

**Table 3:** Primers used for amplification and cloning of *ef0404, ef0648, ef0655* and *ef1181* in pQE30 plasmid

Targeted gene	Primers	Tm (°C)	PCR amplicon
*azoA*	cgggatccTCAAAATTATTAGTTGTTAAAGCACATCC	59.5	644 pb
	acgcgtcgacATTTAGAATGTTTTACCGTATTCAGTTGC	60.4	
*ef0404*	cgggatccACAACATATACAACGAATGATTTTTCAG	59.0	660 pb
	acgcgtcgacTTTTATTGCCTATTCAAATGTCGTG	59.5	
*ef0648*	cgggatccATGTATCAAGATGTTGTTCGCAGC	60.6	701 pb
	acgcgtcgacCAATCACTTTGGATGTTTGTTCC	58.6	
*ef0655*	cgggatccTCAAAATTTACTGAAATGATGAAAAACC	60.1	652 pb
	acgcgtcgacGCTTTCACTCCTTTCCTCTTCG	59.7	
*ef1181*	cgggatccAATCAAACAATCGAACAATTACTAAGTC	57.7	763 pb
	acgcgtcgacCACGCTCTTTTGTTTAGACATC	58.2	

For each gene, primer couples are reported. Nucleotides identical to the gene sequence are in capital letters and nucleotide motifs required for cloning containing restriction sites BamHI or SalI are in lowercase

The purified fragments and the expression vector pQE30 were digested by restriction enzymes BamHI and SalI prior to ligation. The ligation was carried out using T4 DNA ligase (Fermentas, Saint-Rémy-lès-Chevreuse, France) under standard conditions.

All the constructed plasmids were verified by sequencing (GATC Biotech, Konstanz, Germany) to confirm the insertion and the absence of mutations in the sequences cloned.

*E. coli* strain XL1Blue was used as a host strain to facilitate overproduction of the different proteins. The recombinant vectors were transformed into XL1Blue cells by electroporation. The recombinant transformants were selected by their ampicillin resistance (100 mg. l^−1^).

### Purification of enzymes

His-tagged recombinant enzymes were purified according to two different processes previously described by Mercier et al. [18]*.* The native method allowed to recover enzymes including bound cofactors. A denaturation/renaturation protocol allowed the isolation of enzymes without cofactors. Excess (unbound) cofactors and imidazole used in the elution step of purification process were eliminated by dialysis.

Whole cells extracts and overexpressed (and purified) recombinant proteins were analyzed using sodium dodecyl sulphate-polyacrylamide gel electrophoresis (SDS-PAGE) according to the method of Laemmli [20].

### Enzymatic assays

Enzymatic activities were assayed with 10 mg. l^−1^ of purified proteins and 100 μM of substrate. Methyl red and 7NCCA are used as substrate for azo and nitro activities. Reaction is followed in 50 mM sodium phosphate pH 7 buffer added with 0.5 mM NAD(P) H, in a 96-well microplate (Greiner, Courtaboeuf, France).

The kinetic analyses were performed using purified proteins incubated at 35 °C while continuously measuring fluorescence development using an Infinite® M200 microplate reader. Absorbance at both excitation and emission wavelengths were quantified in order to evaluate potential quenching effects.

Nitroreductase activity was evaluated by fluorescence increase at 365/440 nm (excitation/emission), corresponding to emergence of the fluorescent products of 7NCCA nitroreduction. Azoreductase activity was evaluated using methyl red as substrate. Reduction of this compound was detected by absorbance at 435 nm and by fluorescence at 250/395 nm (excitation/emission), parameters used to detect anthranilic acid. All experiments were independently reproduced three to five times. All the fluorescence results were expressed in relative units. To simplify the graph, one experiment in each case has been chosen to draw the curves but all our experiments have shown very good reproducibility.

## Results

### Nitroreductase activity of *E. faecalis* strains

**Fig. 1:**
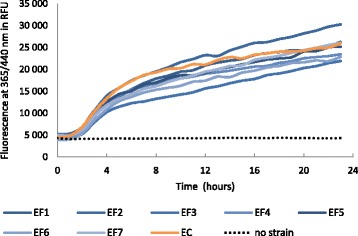
Nitroreductase activity of *E. faecalis* strains. Kinetics of reduction of 7NCCA by 8 different *E. faecalis* strains (EF) and one *E. coli* strain (EC) followed as variation of fluorescence intensity at 365/440 nm in relative fluorescence unit (RFU) over 24 h. *E. coli* strain is used as positive control for nitroreductase activity

In the combined presence of bacteria and the nitroreductase substrate 7NCCA, an increase of fluorescence was observed (Fig.1). All strains showed equivalent growth during this incubation (data not shown). All *E. faecalis* strains showed activity towards this substrate, with limited variation between strains and equivalent to the *E. coli* strain tested as positive control, suggesting a consistent presence of this activity among *E. faecalis* strains.

### In silico search of nitroreductases in the *E. faecalis* V583 genome

Using the NfsA sequence, one putative NADPH-dependent oxidoreductase homologue was found in *E. faecalis* V583 genome: EF1181 (AAO80980.1) showing 56% amino acid sequence similarity. Using the NfsB sequence, two more putative nitroreductases were identified: EF0404 (AAO80264.1.) and EF0648 (AAO80473.1.) with 44 and 37% sequence similarity, respectively. These three proteins were already annotated as possible nitroreductases in the Uniprot database. In this database, another protein was identified as a putative nitroreductase: EF0655 (AAO80478.1).

The Blast search on V583 proteins using AzoR as reference sequence was also performed. Apart from AzoA (AAR38851.1) which shares 58% similarity to AzoR, no additional putative azoreductase was found.

### Phylogenetics of *E. faecalis* azoreductases and putative nitroreductases

**Fig. 2:**
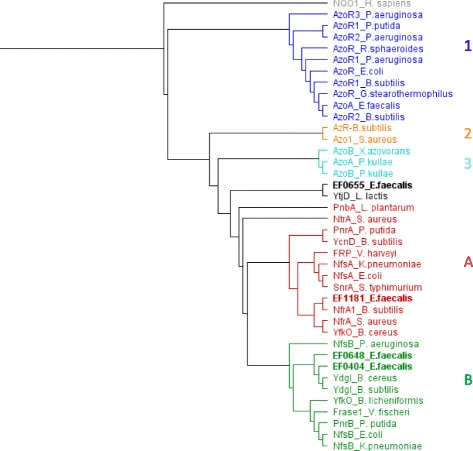
Phylogeny of azoreductase and nitroreductase proteins. Phylogenic tree was generated using a multiple alignment of proteins (Muscle alignment) which have experimentally shown azoreductase and/or nitroreductase activity. Nitroreductases of *E. faecalis*, studied here, are indicated in bold. A specific colour is attributed to each reductases family. For azoreductases, families type 1 to 3 are indicated. In dark blue, type 1 gathers FMN-dependent NADH azoreductases. In orange, type 2 regroups FMN-dependent NADPH azoreductases. In turquoise, type 3 are FMN-independent azoreductases. For nitroreductases, in red, group A represents oxygen-insensitive NADPH dependent nitroreductase. In green, group B gathers oxygen-insensitive NAD(P)H nitroreductase. The nomenclature is the following: first the protein name and then the species. The references of the protein sequences aligned are the following: Azo1_*S. aureus*:AAT29034.1; AzoA_*E. faecalis*:AAR38851.1; AzoA_*P. kullae:*AAO39146.1; AzoB_*X. azovorans*:AAM92125.2; AzoB_*P. kullae*:ADD80733.1; AzoR_*R. sphaeroides*:ABA81336.1.; AzoR_*E. coli*:AAC74494.1.; AzoR_*G. stearothermophilus*:AAD24436.1.; AzoR1_*P. putida*:AAN68474.1.; AzoR1_*P. aeruginosa*:AAG04174.1; AzoR1_*B. subtilis*:CAB13815.1.; AzoR2_*P. aeruginosa*:AAG05350.1; AzoR2_*B. subtilis*:CAB15359.1.; AzoR3_*P. aeruginosa*:AAG06611.1.; AzR_*B. subtilis*:CAB12762.1.; EF0404_*E. faecalis*:AAO80264.1.; EF0648_*E. faecalis*:AAO80473.1.; EF0655_*E. faecalis*:AAO80478.1; EF1181_*E. faecalis*:AAO80980.1; Frase1_*V. fisheri*:BAA04595.2.; FRP_*V. harveyi*:AAA21331.1.; NfrA_S. aureus:ABD29532.1.; NfrA1_*B. subtilis*:CAB15837.1.; NfsA_*K. pneumoniae*:ABR76318.1.; NfsA_*E. coli*:AAC73938.1.; NfsB_*P. aeruginosa*:AAG08575.1.; NfsB_*E. coli*:AAC73679.1.; NfsB_*K. pneumoniae*:ABR76003.1.; NtrA_*S. aureus*:ABD29959.1.; PnbA_*L. plantarum:*CCC77618.1.; PnrA_*P. putida*:AAM95986.1.; PnrB_*P. putida*:AAM95987.1.; SnrA_*S. typhimurium*:AAD18027.1.; YcnD_*B. subtilis*:BAA09018.1.; YdgI_*B. cereus*:AAP09971.1.; YdgI_*B. subtilis*:BAA19399.1.; YfkO_*B. cereus*:AAP08598.1.; YfkO_*B. licheniformis*:EWH23016.1.; YtjD_*L. lactis*:AAK06011.1.; NQO1_*H. sapiens*:AAB60701.1

We aligned the sequences of AzoA and the new putative nitroreductases here identified with previously characterised azo and nitro reductases proteins from different bacterial species and a phylogenetic tree was constructed (Fig 2).

EF1181 harbours a sequence close to that of NADPH-dependent nitroreductase, also indicated as group A (red in Fig. 2). EF0648 inserts into the group of NAD(P)H-dependent nitroreductases (Group B; green in Fig. 2). This group gathers proteins which can use both NADH and NADPH as cofactors for nitroreduction. EF0404 classifies closely to EF0648 among the nitroreductases of group B. EF0655 appears to be distant from nitroreductases of group A and group B, but is similar, at 72%, to YtjD from *Lactococcus lactis* [21]. These two enzymes regroup into the nitroreductase 4-sub family based on amino acids from conservative domains (Conserved Domains Database, NCBI, [22]).

Thus, the four putative nitroreductases identified in *E. faecalis* strain V583 regroup into three different nitroreductase families, with the separation being based on their sequence similarities.

Finally, AzoA, characterised as an azoreductase in *E. faecalis*, is aligned with group 1 (blue in Fig. 2) corresponding to characterised azoreductases, in which some have already been shown to display nitroreductase activity (such as AzoR from *E. coli*) [18].

### Cloning, overproduction and purification of AzoA, EF0404, EF0648, EF0655 and EF1181 proteins

All the previously identified genes encoding proteins AzoA, EF0404, EF0648, EF0655 and EF1181 were successfully cloned in pQE30, which allows for an N-terminal Histidine Tag (His-tag) to be inserted. By sequencing, the inserted sequences were verified: all constructs corresponded to the expected sequences without any mutation present. All the constructs enabled the overproduction and purification of the expected recombinant proteins using His-tag affinity chromatography. On denaturing SDS-PAGE, a unique band was observed for each recombinant protein, approximatively 22 kDa for EF0655, 25 kDa for AzoA, EF0404, EF0648 and 30 kDa for EF1181. These results match the expected molecular weight based on gene sequences and the His-tag motif addition.

As previously described [15, 23, 24], the purified and native recombinant proteins were all yellow, suggesting the presence of protein bound FMN [3]. Liquid Chromatography – Electrospray Ionisation – Mass Spectrometry (LC-ESI-MS) confirmed FMN presence for the enzymes purified using native conditions. When using the denaturation/renaturation protocol [18], which leads to cofactor detachment, the recombinant proteins fractions were colourless and the removal of almost all FMN was demonstrated by LC-ESI-MS analysis. On average, FMN peak intensity was reduced by at least 90% using this purification protocol (data not shown).

### Nitroreductase activity of *E. faecalis* proteins and cofactor preference

**Fig. 3:**
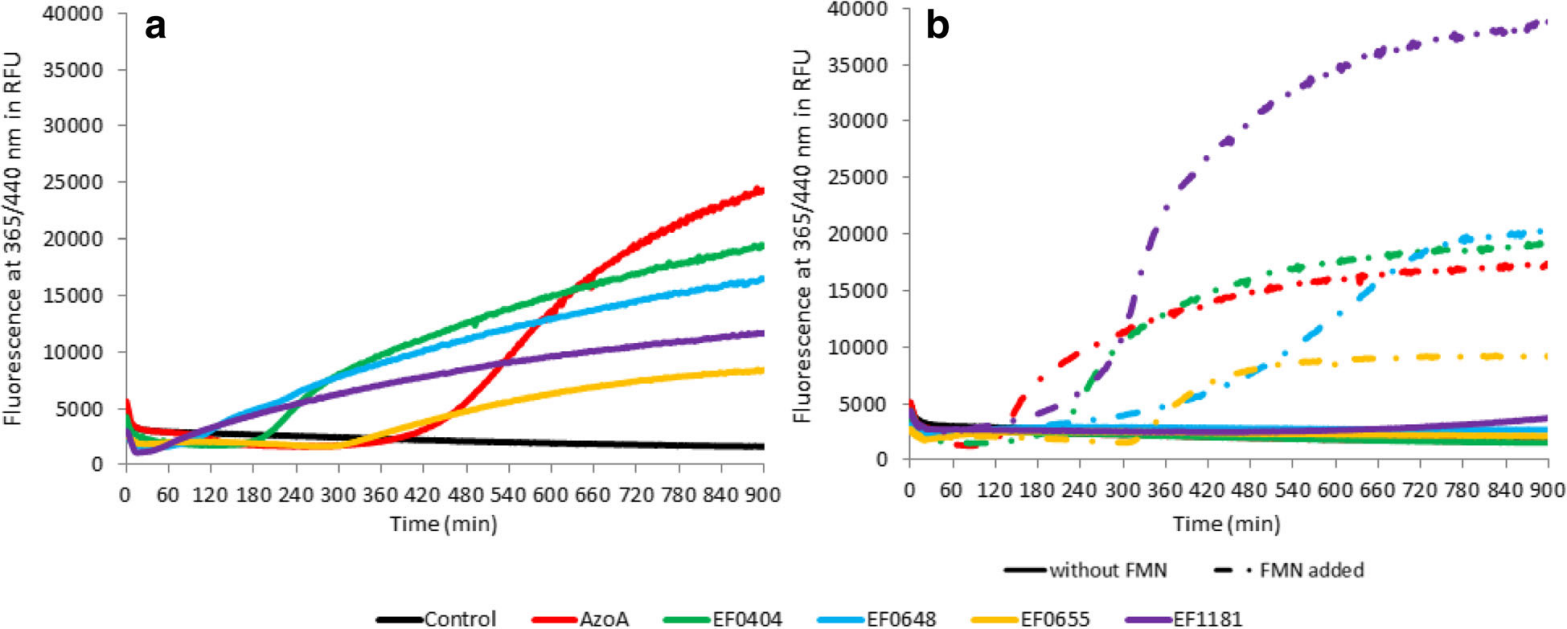
Nitroreductase activity of AzoA, EF0404, EF0648, EF0655, EF1181: native (**a**) and without prosthetic group enzymes (**b**). Nitroreductase activity is evaluated by following fluorescence intensity at 365/440 nm during 900 min in presence of 100 μmol.l^−1^ 7NCCA, 500 μmol.l^−1^ NADPH and 10 mg.l^−1^ of indicated enzyme. **a** The proteins used were purified with their prosthetic group in their native form. No FMN was added for the reaction. **b** The proteins used were purified without their prosthetic group, that is purified using the denaturing/renaturing protocol. The reduction is followed without addition of FMN (−) and with 5 μmol.l^−1^ of FMN (− •)

All of the five recombinant proteins purified under native conditions were tested with 7NCCA as nitro substrate. They were all able to reduce this substrate indicating their nitroreductase activity, as the azoreductase AzoA (Fig. 3a).

For the enzymes without prosthetic group obtained through the denaturation/renaturation protocol, no 7NCCA reduction was observed. Addition of FMN restored the reduction activity for all five recombinant enzymes as shown by the increase of fluorescence (Fig. 3b). Clearly, no fluorescence was observed in the absence of enzyme. Consequently, all the newly identified proteins have now confirmed nitroreductase activity, and so has AzoA, in a FMN-dependent manner.

Nitroreductases are separated regarding their preference toward NADH or NADPH, a cofactor required for electronic exchange to happen [25, 26]. For these five recombinant proteins, we determined which cofactor enabled better 7NCCA reduction, leading to cofactor preference.

**Fig. 4:**
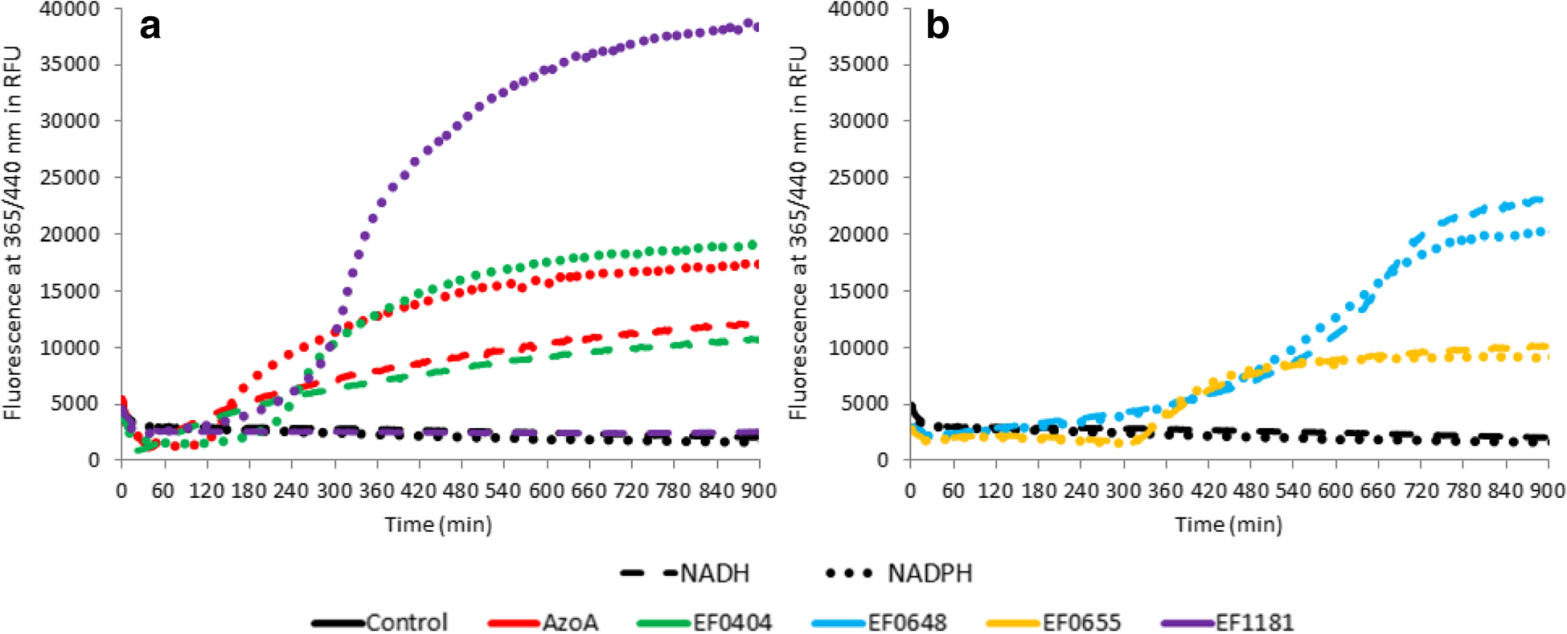
Nitroreductase activity of AzoA, EF0404, EF0648, EF0655, EF1181 and their cofactor preferences. **a**-**b** The proteins used were purified without their prosthetic group, that is purified using the denaturing/renaturing protocol. Nitroreductase activity is evaluated by following fluorescence intensity at 365/440 nm during 900 min in presence of 100 μmol.l^−1^ 7NCCA and 10 mg.l^−1^ of indicated enzyme. To determine each enzyme preference toward cofactors, 7NCCA reduction is followed in presence of 500 μmol.l^−1^ NADPH (• • •) or NADH (− −). FMN is added at 5 μmol.l^−1^. The control wells contained the reaction buffer (7NCCA, FMN, NAD (P) H) without enzyme

EF0648 and EF0655 reduced the substrate equally well in the presence of either NADH or NADPH (Fig 4b), but emergence of fluorescence was delayed compared to the other proteins. However, it is worth noting that fluorescence was detected earlier when native EF0648 was used for nitroreduction (Fig 3a), which indicates that purifying this enzyme without prosthetic group might affect its resultant conformation. For EF0655, similar results were obtained with both purification protocols. The fluorescence delay might be due to protein conformation issues, and indicates a weaker activity compared to EF0648.

With EF0404, an increase of fluorescence was observed in the presence of NADH and NADPH, suggesting that this enzyme can use both cofactors (Fig. 4a). However, fluorescence slopes indicated a preference towards NADPH.

Interestingly, AzoA, the azoreductase which is able to reduce the nitro substrate, showed very similar results when compared to EF0404 for 7NCCA reduction. AzoA was able to use both NADH and NADPH as cofactor, with an increased activity with the latter (Fig. 4a).

EF1181 was able to reduce the 7NCCA using NADPH only (Fig. 4a). This is evidence for EF1181 being a strictly NADPH-dependent nitroreductase. EF1181 nitroreduction was higher for the enzyme purified using denaturation/renaturation protocol with FMN addition than for the native form (Fig. 3). This suggests that EF1181 purified in its native form might lack of FMN to exhibit full activity.

We showed that all five purified proteins share nitroreductase activity while having different cofactor specificities.

### Azoreductase activity of *E. faecalis* proteins and cofactors preference

**Fig. 5:**
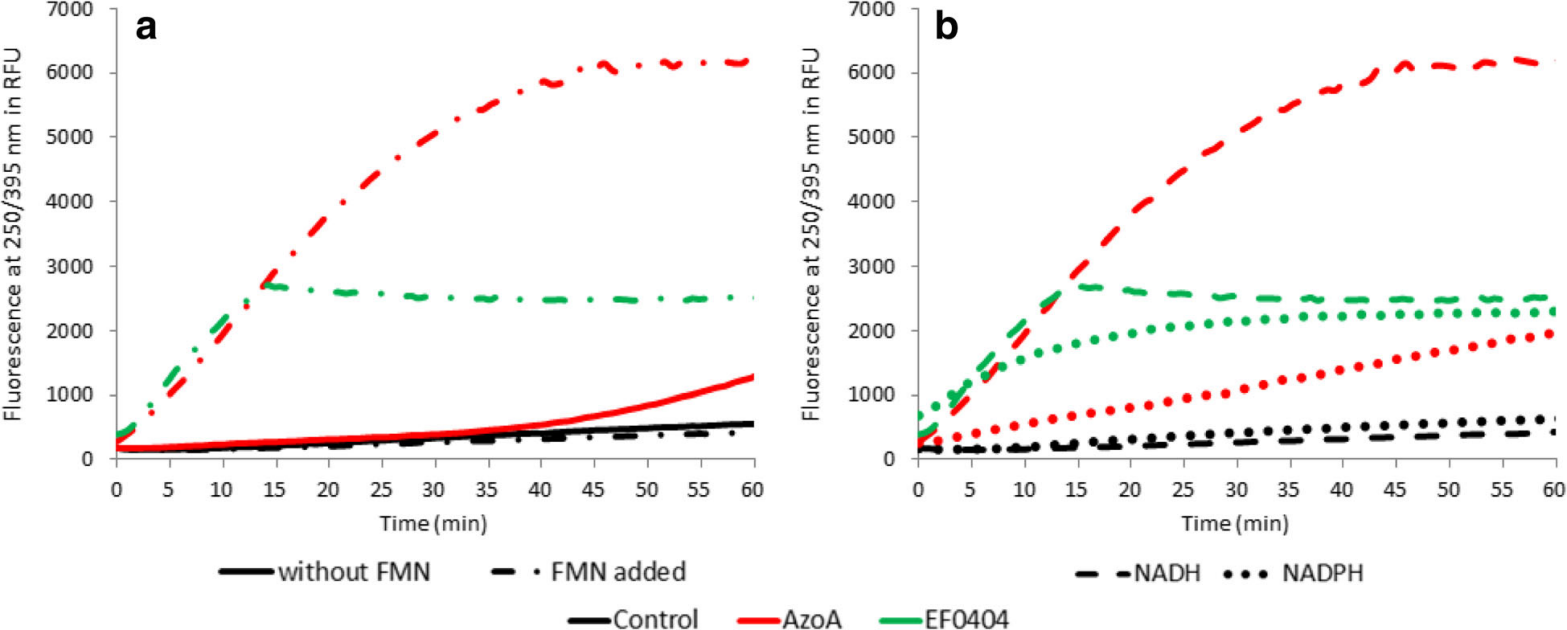
Azoreductase activity of AzoA and EF0404 and their cofactor preferences. The proteins used were purified without their prosthetic group, that is purified using the denaturation/renaturation protocol. For the enzymes presenting azoreductase activity, methyl red reduction is monitored by fluorescence intensity at 250/395 nm during 60 min in presence 10 mg.l^−1^ of indicated enzyme. **a** To test the importance of FMN for these enzymes, methyl red (100 μmol.l^−1^) reduction is followed without addition of FMN (**−**) and with 5 μmol.l^−1^ of FMN (**−** •). NADPH is added at 500 μmol.l^−1^. **b** Methyl red (100 μmol.l^−1^) reduction is followed in presence of 500 μmol.l^−1^. NADPH (• • •) or NADH (− −) to evaluate cofactors preference. FMN is added at 5 μmol.l^−1^. The control wells contained the reaction buffer (methyl red, FMN, NAD (P) H) without enzyme

AzoA is able to reduce methyl red as demonstrated by monitoring the formation of the fluorescent end-product anthranilic acid. Among the nitroreductases identified in this work, EF0404 was also able to reduce this azo substrate (Fig. 5a and b). For the three others nitroreductases EF0648, EF0655 and EF1181, no azoreductase activity was detected with methyl red, as no substrate reduction was observed whatever cofactor was used (NADH or NADPH, data not shown).

Without FMN addition, EF0404 purified without prosthetic group was unable to reduce the methyl red, indicating its FMN-dependence for azoreductase activity. In case of AzoA, without FMN addition, a late and slight fluorescence increase was observed. This result might be due to residual bound or unbound FMN within the protein solution.

For both EF0404 and AzoA, cofactor preference was studied (Fig. 5b). Both proteins showed increased azoreduction in the presence of NADH. For EF0404, azoreduction of methyl red in presence of NADPH is low. Even if NADPH presence can lead to methyl red reduction by EF0404 and AzoR, fluorescence pattern seems to indicate that NADH is the natural cofactor for this activity.

## Discussion

The major aim of this work was to detect and confirm *E. faecalis* nitroreductase activity and to further characterise the enzymes which are responsible for this activity. First, overall nitroreductase activity was demonstrated for a panel of *E. faecalis* strains. Secondly, the four putative nitroreductases identified using BLAST research on *E. faecalis* V583 genome and the azoreductase AzoA were cloned, expressed and purified. When purified without FMN, the enzymes were unable to reduce the substrates tested, confirming their FMN-dependence.

EF0404, EF0648, EF0655 and EF1181 are the first experimentally confirmed nitroreductases in *E. faecalis* and each enzyme presented specific cofactor dependence (Table 4). As nitroreductases are distinguished by their preference towards NADH or NADPH [8], there was an interest to define the phylogeny of the newly discovered enzymes. EF1181 was only able to reduce the nitro substrate using NADPH as cofactor which correlates with its position in NADPH-dependent nitroreductases group, also known as group A. Thus, EF1181 might be the *E. faecalis* equivalent of NfsA in *E. coli.* EF0648 is part of the nitroreductases group B which can use both NADH and NADPH as cofactors as shown by our experiments. Hence, EF0648 might be the equivalent in *E. faecalis* of NfsB in *E. coli*.

**Table 4:** Summary of purified proteins activities

	Enzymes families	Reductase activity	FMN-dependence	NADPH	NADH
AzoA	Type 1: FMN-dependent NADH azoreductase	Azo +	+	+	++
		Nitro +	+	++	+
EF0404	Group B: FMN-dependent NAD(P)H nitroreductase	Azo +	+	+	++
		Nitro +	+	++	+
EF0648	Group B: FMN-dependent NAD(P)H nitroreductase	*Azo -*	*NA*	*NA*	*NA*
		Nitro +	+	+	+
EF0655	FMN-dependent Nitroreductase	*Azo -*	*NA*	*NA*	*NA*
		Nitro +	+	+	+
EF1181	Group A: FMN-dependent NADPH nitroreductase	*Azo -*	*NA*	*NA*	*NA*
		Nitro +	+	++	−

For each enzyme, it is reported whether it presents nitroreductase and/or azoreductase activity: +: Activity has been established by reduction of tested compound; −: No activity was observed; NA: not applicable

When activity was established, FMN-dependence and cofactor dependence/preference are indicated

While EF1181 and EF0648 results correlated well to phylogenetic classification, EF0404 results were more intriguing. Indeed, EF0404 was closely related to EF0648 among nitroreductases of group B, but EF0404 reduced both the azo and nitro substrates tested. Of both enzymes, only EF0404 reduced methyl red. Moreover, for 7NCCA reduction, EF0404 demonstrated a preference for NADPH, which is not the case for EF0648 (Table 4). Therefore, regarding activity results, EF0404 didn’t appear close to EF0648. To our knowledge, no nitroreductases from group A have been shown to be able to reduce azo compounds directly. *E. coli* nitroreductases, NfsA and NfsB, can reduce azo compounds but only indirectly, in a lawsone (2-hydroxy-1,4-naphthoquinone) dependent manner [27]. It is NfsA and NfsB ability to reduce lawsone into hydroquinone that leads to further chemical reduction of the azo compound. Here, EF0404 reduced methyl red directly with no addition of redox mediators, confirming azoreductase activity of this enzyme. Consequently, for EF0404, the results obtained were much closer to those obtained with the azoreductase AzoA, although their structure and similarities were quite distant.

Interestingly, a single amino acid substitution in the active site of the azoreductases in *E. coli*, *E. faecalis* or *Pseudomonas aeruginosa* was shown to modify substrate specificity, cofactor binding or activity [28–30]. Consequently, we can hypothesize that one or more amino acid changes in the EF0404 active site might explain its ability to reduce methyl red whereas the other nitroreductases tested cannot. There are two established motifs defining amino acids for FMN binding and dimer interface in nitroreductase. EF0404 is presenting four amino acids different from the consensus for FMN binding (which are identical in EF0648) and which appear to affect protein structure. Both proteins presents more difference toward the consensus for dimer interface and none of these differences modify the protein structure elements. Differences in binding FMN might then modify the panel of substrates.

Azoreductases have already been described to actively modify nitro compounds. For example, AzoR in *E. coli* is able to reduce CB1954 [6] as well as the 7NCCA used here [18]. In agreement with previous results, we demonstrate here that AzoA from *E. faecalis* is capable of nitroreduction. The reduction of nitro compounds by AzoA is probably based on the same mechanism as was shown for AzoR1 of *P. aeruginosa* with nitrofurazone [31].

Finally, EF0655 appears to be distant from nitroreductases of groups A and B and shares 59% identity with YtjD from *Lactococcus lactis* [21]. EF0655 and YtjD are 63% and 58% homologous to the nitroreductase 4 family consensus sequence, respectively. YtjD was studied in detail since its activity is regulated by copper. Genetically, no similarity was found between *ef0655* and *ytjD* and therefore no regulatory regions were identified in *ef0655.* Moreover*, ef0655* was not shown to be affected by copper in transcriptomic studies [32]*.* However, an *E. faecalis* metabolic networks have shown highly conserved connections within the *Lactobacillales* order when exposed to copper [33]. Therefore EF0655 and YtjD might be inherited from a common *Lactobacillales* ancestor [34]. Consequently, it might be of interest to test copper-mediated induction of *ef0655*. EF0655 is a nitroreductase, which *in cellulo* role might differ from the one of EF0648 and EF1181. In fact, this enzyme had the lowest and most delayed activity on the nitro substrate tested.

Separation of enzymes based on their sequence homology tends to exclude the possibility of these enzymes to have different reductase activities. For example, it was recently shown that MdaB, ArsH and YieF from *P. aeruginosa* can reduce different azo compounds while being part of distantly homologous oxido-reductases families with respect to protein sequence. Interestingly, these proteins were also proven to reduce quinones and nitrofurazone [35]. Consequently, biochemical assays are clearly necessary to corroborate the protein homologies.

Previously, azoreductases were shown to better reduce quinones than azo compounds. Because of this observation and the related reaction mechanism, it is already suggested that azoreductases and quinone reductases have a common physiological role and group into the same enzymatic families [36]. Nitroreductases are also able to reduce quinones, sometimes with higher affinity than for nitro compounds [27, 37]. According to the results we obtained with AzoA and EF0404, we emphasize the abilities of azoreductases and nitroreductases to complement each other. Considering, azoreductases, nitroreductase and quinone reductases as one group of enzymes could help to understand their role in the bacterial cellular mechanisms.

## Conclusions

Diverse *E. faecalis* enzymes belonging to different oxido-reductase families are able to reduce the same nitro compound. Our work clearly demonstrate that the experimental proof of activity is necessary to identify the substrate specificity of each enzyme as homologies with other known reductases is not sufficient. The redundancy of reductase in *E. faecalis* may be an indication that such activities are important. It could also indicate that each of these enzymes may have a preferred domain of activity depending on the environment and/or on the availabilities of substrates and cofactors. Both hypotheses should be taken into consideration to identify enzymes for processes or therapies that would depend on these kind of activities, such as for the bioremediation of azo dyes or the use of nitroaromatic drugs.

## Abbreviations

7NCCA: 7-nitrocoumarin-3-carboxylic acid
EC: *Escherichia coli*
EF: *Enterococcus faecalis*
FMN: Flavin mononucleotide
LC-ESI-MS: Liquid chromatography – electrospray ionisation – mass spectrometry
NADH: Nicotinamide adenine dinucleotide hydrogen
NADPH: Nicotinamide adenine dinucleotide phosphate hydrogen
SDS-PAGE: Sodium dodecyl sulphate-polyacrylamide gel electrophoresis
TNT: 2, 4, 6-trinitrotoluene
